# Structure-Activity Studies on the Hypertrehalosemic Hormone II of the Stick Insect *Carausius morosus* (Phasmatodea): Carbohydrate-Mobilization and Cardio-Stimulatory Activities

**DOI:** 10.3389/fphys.2020.00315

**Published:** 2020-04-28

**Authors:** Ottilie K. H. Katali, Heather G. Marco, Gerd Gäde

**Affiliations:** Department of Biological Sciences, University of Cape Town, Rondebosch, South Africa

**Keywords:** adipokinetic hormone, hypertrehalosemic hormone, fuel mobilization, heart beat rate, *Carausius morosus*, Carmo-HrTH-II, structure-activity studies, stick insect

## Abstract

The corpora cardiaca of the Indian stick insect, *Carausius morosus*, synthesize two decapeptide neuropeptides of the adipokinetic hormone (AKH) family, both of which can increase the trehalose levels in the hemolymph when the stick insect is ligated between the head and the thorax. Here, we use two biological assays to assess the potencies of 19 AKH analogs in ligated *C. morosus*: the carbohydrate-mobilizing assay measures the change in the levels of circulating carbohydrates following injection of a substance, while the semi-exposed heart assay measures a change in heart beat rate after the peptide is applied onto the heart. With the endogenous AKH (Carmo-HrTH-II) as lead peptide, we report here on seven naturally-occurring AKH peptides (bioanalogs) selected for testing because of a single or double amino acid replacement, or for being octapeptides. Single amino acid substitutions by an alanine residue at all positions of Carmo-HrTH-II, as well as analogs modified at the termini were also investigated to give a comprehensive view of ligand-receptor interaction at the physiological level in a hemimetabolous insect that practices thanatosis (feigning death). Only small changes are elicited in the bioassays, but the results from the two tests are comparable bar one or two anomalies. Results show that analogs modified at the termini have no or reduced activity. Regarding structural requirements of a ligand, the *C. morosus* AKH receptor appears to be strict: octapeptides are not preferred and many of the decapeptide analogs failed to reach 50% activity relative to Carmo-HrTH-II. The data implies that the AKH receptor in *C. morosus* mostly does not tolerate shorter peptides and single amino acid replacements in most places of the native AKH peptide. This information is important if environmentally friendly insect-specific pesticides are made based on an insect AKH as lead peptide: stick insects that are normally not viewed as pest insects may not be easily targeted by cross-reactive AKH mimetics directed at harmful insects, due to the very specific amino acid requirements to activate the *C. morosus* AKH receptor.

## Introduction

Insects are not only well-known for their diversity and abundance but also for their influence on the biosphere and human life. The major anthropomorphic division in insect groups is made between those that are beneficial to mankind and those that are health risks or agricultural pests (Burn et al., 1987; Capinera, 2010; Gullan and Cranston, 2014). Increasingly, there is a strong interest in developing environmentally friendly insecticides that are selective and affect only the target (pest) species instead of all insects. Specifically, the development of hormone-like compounds that can be used in specific drug design to act as targeted pesticides are being considered (Altstein et al., 2000; Gäde and Goldsworthy, 2003; Audsley and Down, 2015). The compounds in mind are the insect’s neuropeptide hormones that control most of the key physiological processes such as development, reproduction, metabolism, behavior, muscle contraction including heart beat rate and diuresis.

A well-researched family of hormones are the adipokinetic hormones (AKHs) which are synthesized and released from the retrocerebral corpora cardiaca (CC); the AKHs are mainly tasked with mobilizing fuels (energy-rich metabolites) from fat body stores into the hemolymph and are identified as putative targets to develop new insecticides (see review by Marco and Gäde, 2020). The AKH peptide family is generally characterized by peptides having (a) a chain length of 8–10 amino acids; (b) post-translationally modified termini: a pGlu residue at the N-terminus and a carboxyamide at the C-terminus; (c) either a Leu, Ile, Val or Phe residue at position 2; (d) a Thr or Asn residue at position 3; (e) an aromatic Phe or Tyr residue at position 4; (f) the branched amino acids Thr or Ser at position 5; (g) the aromatic residue Trp at position 8; (h) the simple amino acid Gly at position 9, and (i) variable amino acids at positions 6, 7, and 10 (see Gäde, 2009).

AKHs exert a biological effect via a G protein-coupled receptor (GPCR) and this system, the ligand-receptor pair, is the target for peptide mimetics to be developed (see reviews by Audsley and Down, 2015; Verlinden et al., 2015) in a fashion similar to the well-known beta blockers treating hypertension in human medicine. Two standard methods have been used to investigate AKH ligand-receptor interactions in structure-activity relationship (SAR) studies; the oldest being an indirect *in vivo* biological assay in which ligands are tested in live animals, and the result of a signal transduction cascade is measured, e.g., the release of lipids/carbohydrates into the hemolymph, or the activation of glycogen phosphorylase. This has been done for AKH bioanalogs and synthetic analogs in locusts (see, for example, Stone et al., 1978; Gäde, 1990, 1993; Poulos et al., 1994; Goldsworthy et al., 1997), lepidopterans (Fox and Reynolds, 1991; Ziegler et al., 1991, 1998; Marco and Gäde, 2015, 2019) and cockroaches (Gäde, 1986, 1990, 1992; Ford et al., 1988; Hayes and Keeley, 1990; Gäde and Hayes, 1995). The second and more recent method of conducting SARs is via a direct *in vitro* receptor assay; the prerequisite is to have knowledge of the AKH receptor sequence: this is in general (with a few exceptions) only the case for those insects where the whole genome is known. To date, detailed receptor assay SAR studies with bioanalogs and specifically modified peptides have only been performed with dipteran species, *Drosophila melanogaster*, *Anopheles gambiae* and *Glossina morsitans* (Caers et al., 2012, 2016). In general, the results of these *in vitro* assays agree with those generated by *in vivo* biological assays, i.e., that the conserved aromatic amino acids at position 4 and 8 are important for receptor-peptide interactions, as well as (in the majority of cases) the blocked termini, whereas amino acids at positions 7 and 10 are not always that crucial. It also appears to emerge that the receptor of those species that have two or more endogenous AKHs, such as *Periplaneta americana*, *Locusta migratoria*, and *Hippotion eson*, seem to tolerate a wider variety of ligand modifications than an insect with a single endogenous AKH, such as *Blaberus discoidalis* and *Aedes aegypti* (see references above; Marchal et al., 2018; Wahedi et al., 2019).

AKHs are also known for their myotropic effects, especially to increase the rate of heart beat (Chowański et al., 2016); SAR studies have only been done in the cockroach *P. americana* to a certain extent (Baumann et al., 1990), and a few bioanalogs were tested on the heart of the stick insect *Baculum extradentatum* (Malik et al., 2012).

In the current study, a member of the order Phasmatodea is investigated with respect to metabolic (hypertrehalosemic) and myotropic (cardio-stimulatory) activity of AKH. Insects of this order are well known to be kept as pets, although there are also reports on the pest status of certain phasmid species, especially in private gardens on ornamental plants in the United States of America (Griffiths and Picker, 2011). The subject of the current study, the Indian stick insect *Carausius morosus*, is already well-known in laboratory research of neuropeptide hormones (see for example Miksys et al., 1997; Predel and Gäde, 1999; Lorenz et al., 2000; Liessem et al., 2018), and has also become a model organism for neurobiology, especially studying the control of locomotion (see for example, Bidaye et al., 2018).

With regards to AKH research on *C. morosus*, two near-identical decapeptide members of the AKH family were isolated from the corpora cardiaca, and these peptides had no effect on hemolymph metabolite levels in conspecific assays (Gäde, 1979). Three years later, a hypertrehalosemic effect of conspecific CC extract was demonstrated in adult *C. morosus* only when a ligature was applied to the insect (between the head and the first pair of legs) before injection of the CC extract (Gäde and Lohr, 1982). The exploratory study of 1982 further revealed that the increase in circulating trehalose was relatively small (about 4–7 mg ml^–1^); the highest response was recorded in ligated 6th instar larvae shortly before the final molt; and there was evidence of ligand preference, i.e., the AKH receptor of the stick insect recognized the conspecific AKHs but not that of lepidopteran species, the migratory locust or of the decapod crustaceans (Gäde and Lohr, 1982). The two stick insect AKHs were sequenced by fast atom bombardment mass spectrometry (FABMS) (Gäde, 1985; Gäde and Rinehart, 1987): the later eluting 2nd peak contained most of the peptidic material and has the code-name Carmo-HrTH-II; the earlier eluting peak (code-name Carmo-HrTH-I) was shown to be identical in the primary sequence to Carmo-HRTH-II but a hexose is bound in an unorthodox manner to the Trp residue at position 8 (Gäde et al., 1992). Through the heroic collection of 2000 CC and the use of a sensitive 800 MHz nuclear magnetic resonance spectrometer equipped with a cryoprobe, it was possible to show unequivocally that an α-mannopyranose is bound to Trp^8^ of Carmo-HrTH-I in an unusual C-glycosylated fashion (Munte et al., 2008). Recently, a transcriptomic and neuropeptidomic analysis of the central nervous system of *C. morosus* revealed only one AKH precursor although both Carmo-HrTH-I and -II were confirmed in mass spectrometry of tissues (Liessem et al., 2018), thus, the one peptide precursor is modified post-translationally to form both versions of *C. morosus* AKHs.

Recently it was also shown that both AKH peptides of *C. morosus* are capable of increasing the rate of heart contraction in ligated stick insects: the application of synthetic Carmo-HrTH-II in doses above 6.67 × 10^–8^ M increased the heart beat rate significantly and maximally (i.e., higher peptide doses had no further increase on the heart beat rate; Marco et al., 2018). The frequency of heart contractions in *C. morosus* at rest was found to be much lower than that recorded in more active insect species and again, the endogenous AKH peptide could not affect heart contractions in non-ligated *C. morosus* while small increases in the heart beat rate were recorded in ligated stick insects (Marco et al., 2018). Prior to this study, an influence of AKH on stimulating the heart frequency of the Vietnamese stick insect, *B. extradentatum*, was also shown in decapitated specimens (Malik et al., 2012). The low heart rate and the small increases in metabolic reactions to endogenous AKHs are interpreted as evolutionary consequences of the cryptic defenses of stick insects where they shut down the metabolism to play dead (thanatosis) to avoid detection from predators, instead of entering into the fight or flight mode (Marco et al., 2018).

Based on the above-mentioned results, the present study was initiated with the following aim: to understand in detail the requirements of the AKH receptor with respect to structural features of the ligand. Although a number of detailed SAR studies have been performed on the AKH system in insects (references see above), there is only one case according to our information where an insect that has one decapeptide as AKH ligand has been tested; this is the cockroach *B. discoidalis* and its AKH Bladi-HrTH (Ford et al., 1988; Hayes and Keeley, 1990). That study and the companion research on *P. americana* (Gäde and Hayes, 1995), an insect with AKH octapeptides, show that the receptors have some different properties. Since the AKH receptor of *C. morosus* was not structurally known at the time of the current study, we performed *in vivo* bioassays and tested for a metabolic response (hypertrehalosemic activity) and a myotropic response (heart beat rate). In this way we also hoped to establish whether the putative receptor in the fat body tissue and in the heart muscle tissue are very likely identical. Bioanalogs and specifically altered peptides were chosen/designed to obtain the following information on structural requirements for interaction with the *C. morosus* AKH receptor: (a) acceptable chain length of the ligand, (b) the importance of the AKH termini, and (c) the side chain of each amino acid in the decapeptide.

## Materials and Methods

### Insects

Indian stick insects (*Carausius morosus)* were reared under crowded conditions at approx. 25 ± 2°C, 65% RH and a 12 h light: 12 h dark regime. The stick insect nymphs were fed with fresh ivy (*Hedera helix)* leaves supplied twice a week, while the adults were provided with fresh twigs of mirror bush (*Coprosma repens*) about twice a week.

### Peptides and Corpora Cardiaca Extract

For sequence information of the various synthetic peptides (see Tables 1–3).

**TABLE 1:** The biological effect (metabolic) of the termini of Carmo-HrTH-II in ligated 6th instar Indian stick insects (*Carausius morosus*).

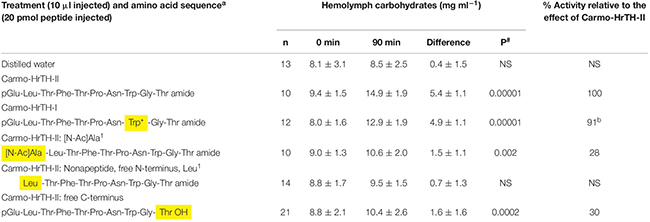

The data are presented as Mean ± S.D. ^*a*^Amino acid substitutions in Carmo-HrTH-II analogs are highlighted. *Tryptophan residue is mannosylated. ^*b*^Not significantly different from the effect of Carmo-HrTH-II (which was set as 100%), as determined by ANOVA and post hoc Scheffe’s test. ^#^Paired t-test was used to calculate the significance between pre-and post-injection values. NS, not significant.

**TABLE 2:** The biological effect (metabolic) of selected AKH bioanalogs in ligated 6th instar Indian stick insects (*Carausius morosus*).

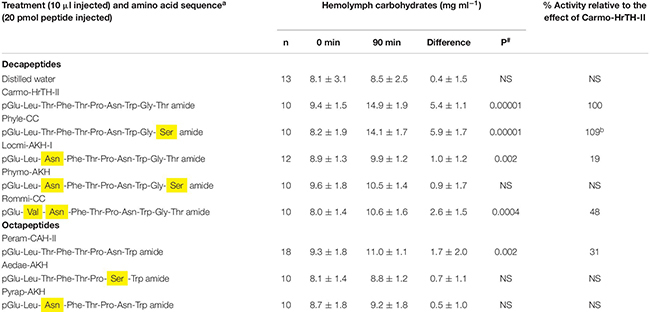

*^*a*^Amino acid substitutions in Carmo-HrTH-II analogs are highlighted. ^*b*^Not significantly different from the effect of Carmo-HrTH-II (which was set as 100%), as determined by ANOVA and post hoc Scheffe’s test. The data are presented as Mean ± S.D. ^#^Paired t-test was used to calculate the significance between pre-and post-injection values. NS, not significant.*

**TABLE 3:** The biological effect (metabolic) of single amino acid substitutions in Carmo-HrTH-II in ligated 6th instar Indian stick insects (*Carausius morosus*).

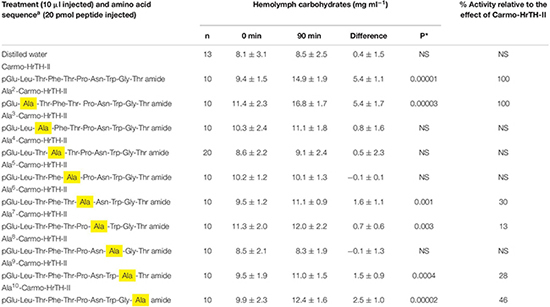

*^*a*^Amino acid substitutions in Carmo-HrTH-II analogs are highlighted. Data presented as Mean ± S.D. ^∗^Paired t-test was used to calculate the significance between pre-and post-injection values. NS, not significant.*

The endogenous hypertrehalosemic hormone Carmo-HrTH-I was isolated from the CC of *C. morosus* as previously described by Gäde (1985). Synthetic Carmo-HrTH-II was purchased from Peninsula Laboratories (Belmont, CA, United States) together with Locmi-AKH-I and Peram-CAH-I. Phyle-CC and Pyrap-AKH were synthesized by Dr. R. Kellner (Merck, Germany). Aedae-AKH was made by Genscript Corporation (United States). Carmo-HrTH-II as free acid and not as amidated form, as well as Carmo-HrTH-II minus pGlu (thus a nonapeptide), and Phymo-AKH were synthesized by Dr. S. Kyin (Biotechnology Centre, University of Illinois, Urbana-Champaign, United States). Analogs of Carmo-HrTH-II with single amino acids replaced by Ala were purchased from Pepmic Co., Ltd (Suzhou, China). Stock solutions were made by dissolving 1 mg of each peptide in 1 ml of 60% acetonitrile, including 0.10% trifluoroacetic acid (TFA), and diluted further to 5 pmol μl^–1^ using distilled water. The various peptide solutions were subsequently monitored for purity and quantified via reversed phase-high performance liquid chromatography (Gilson RP-HPLC system) with fluorescence detection (276 nm excitation, 350 nm emission; Gäde, 1985) and with a gradient of 43–53% solvent B in 20 min at a flow rate of 1 ml min^–1^ (Nucleosil C18 column; Solvent A: 0.11% TFA in water; solvent B: 60% acetonitrile with 0.10 TFA).

Corpora cardiaca (with the corpora allata attached) were dissected from the head of adult *C. morosus* into 80% methanol, the cell contents were extracted on ice via sonication (Branson sonifier cell disruptor), followed by centrifugation and the resulting supernatant was dried in a vacuum concentrator. The dried corpora cardiaca extracts were then reconstituted in distilled water for use in carbohydrate-mobilization assays.

### Bioassays

#### Carbohydrate-Mobilization Assay

Sixth instar nymphs that were 1–2 days before molting were neck-ligated and used in the hypertrehalosemic *in vivo* assay as described in detail by Gäde and Lohr (1982). Stock peptide solutions (5 pmol μl^–1^) were diluted with distilled water to the desired concentration in 10 μl, which was injected into the stick insect nymphs.

#### Semi-Exposed Heart Bioassay

The semi-exposed heart bioassay was carried out as described (Marco et al., 2018) with adult stick insects between 1 and 2 months old that were ligated at the neck. Briefly, a ventral incision of the abdomen exposed the ventral cavity with the internal organs; a few drops of stick insect saline (pH 6.6; 15 mM NaCl, 18 mM KCl, 7.5 mM CaCl_2_, 2 mM HEPES, 50 mM MgCl_2_, and 184 mM glucose according to Ejaz and Lange, 2008) were added to the exposed part. The semi-isolated heart preparation was viewed with a dissecting microscope (12-fold magnification), and the number of observed heart beats (contractions of the heart muscle) was counted during a fixed period using a manual tally counter and a timer. Once the heart had stabilized, saline was replaced with the test solution. For this the peptide stock solution of 5 pmol μl^–1^ was diluted with stick insect saline to the desired concentration and a final volume of 150 μl was used for each test.

Heart rates under normal saline served as controls. The change in heart rate after the application of the test solution was calculated as an average of the first four counts and was compared to the average heart rates counted in saline before the application of the test solution. This comparison of change in heart rates was done for all the peptides tested.

### Statistical Analyses

Student’s paired *t*-test was used to compare the concentrations of carbohydrates in the hemolymph, as well as the heart rates of *C. morosus* before and after the subjection to the test solution. One-way analysis of variance (ANOVA) followed by Scheffe’s multiple comparison test was used to compare the hypertrehalosemic and cardio-stimulatory effects among *C. morosus* tested with different peptides. Differences were considered significant at *p* < 0.05 for all the tests.

## Results

### Hypertrehalosemic Effects of the Endogenous Peptides

**FIGURE 1:**
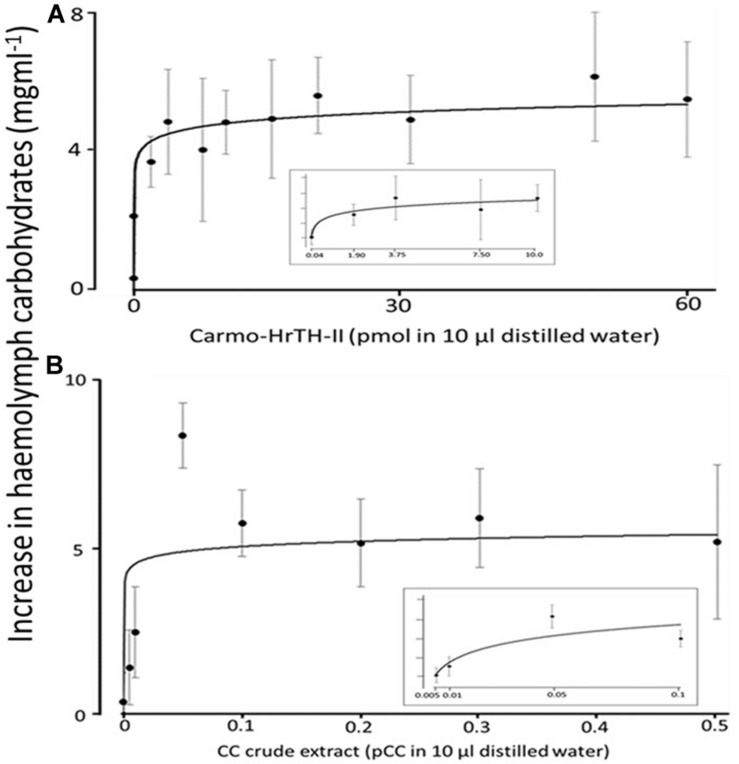
*In vivo* hypertrehalosemic assays. The effects of the increasing doses of **(A)** synthetic Carmo-HrTH-II and **(B)** native CC extracts, on carbohydrate mobilization in ligated 6th instar nymphs of *C. morosus* (17–18 days old). Each data point represents the mean ± SD in mg ml^–1^. Between 7 and 12 insects were used for each dose.

In the first series of experiments, various doses of synthetic Carmo-HrTH-II and corpora cardiaca (CC) extract of *C. morosus* were used to determine the maximal hypertrehalosemic response of *C. morosus.* Hemolymph samples were taken from 17 to 18 days old 6th instar nymphs 2 h after they were neck-ligated and then 90 min after either CC extract or synthetic Carmo-HrTH-II was injected. Ligated nymphs injected with distilled water served as controls for handling stress. As depicted in Figures 1A,B, various doses of both Carmo-HrTH-II and CC extract increased the level of carbohydrates in the hemolymph in a dose-dependent manner. A dose of 0.04 pmol of synthetic Carmo-HrTH-II and the equivalent of 0.005 pairs of corpora cardiaca (pCC) were sufficient to give significant increases in hemolymph carbohydrates (*p* < 0.05). With synthetic Carmo-HrTH-II, doses of 7.5–60 pmol gave an average increase of 5.2 ± 0.4 mg ml^–1^ and the *post hoc* Scheffe’s test revealed that there was no significant difference between the effect of these doses (*p* > 0.05). Doses of 0.1–0.5 pCC gave an average increase of 5.6 ± 0.6 mg carbohydrates ml^–1^ hemolymph (Figure 1B); again, statistical analyses reveal no significant difference between the effect of these doses (*p* > 0.05). An anomalous result was obtained after injection with 0.05 pCC extract: a much higher increase in carbohydrate concentration (8.5 ± 1.0 mg ml^–1^) was measured than from any other dose tested (Figure 1B), and this increase differed significantly (*p* < 0.05) from all the higher CC extract doses; moreover, such an increase was not achieved by any injection of synthetic Carmo-HrTH-II even at high doses (see Figure 1A).

Based on these pilot experiments, we selected a dose of 20 pmol for testing the analogs of Carmo-HrTH-II since such a dose of our lead compound, Carmo-HrTH-II had achieved maximal hypertrehalosemic activity.

### Hypertrehalosemic Response of Synthetic Analogs of Carmo-HrTH-II

#### The Biological Effect of the Termini of Carmo-HrTH-II in *C. morosus*

AKH peptides are characterized by a pGlu in position 1 and an amidated C-terminus. This is believed to be an effective block against exopeptidases in the hemolymph of the insect, resulting in a longer half-life of the peptide (Oudejans et al., 1996). Carmo-HrTH-II analogs were designed to specifically explore how carbohydrate mobilization is affected by changes to the N terminal amino acid of the decapeptide. The results are shown in Table 1. When the termini of Carmo-HrTH were modified (i.e., N-terminal pGlu replaced with an acetylated Ala residue, or a free acid at the C-terminus instead of an amidation) the hypertrehalosemic activity was severely reduced from 100% to a mere 30%, while a nonapeptide lacking a pGlu and having, thus, a Leu residue in position 1 had no significant biological effect.

#### The Biological Effect of AKH Bioanalogs in *C. morosus*

Functional AKHs are either composed of eight, nine, or 10 amino acids; *C. morosus* synthesizes only decapeptide AKHs. The biological effect of shorter chain lengths (octapeptides) were therefore investigated, along with single or double amino acid substitutions in naturally occurring decapeptide AKHs to ascertain the flexibility of the *C. morosus* receptor that usually sees and responds only to two decapeptides of the same amino acid sequence.

Peram-CAH-II has the same 8 amino acids as in the endogenous decapeptide of the stick insect, Carmo-HrTH; this is, however, only sufficient to elicit a 30% hypertrehalosemic response (Table 2). The remaining octapeptides tested with single substitutions at position 3 and 7 had no significant activity.

Of the decapeptide bioanalogs, only Phyle-CC (Ser^10^ instead of Thr^10^) increased the hemolymph carbohydrates as high as Carmo-HrTH-II (*p* > 0.05), while Rommi-CC (Val^2^-Asn^3^ instead of Leu^2^-Thr^3^), elicited a response of 48% of the maximal possible hypertrehalosemic effect. The remaining analogs were virtually unable to increase the hemolymph carbohydrates (Table 2).

#### The Biological Effect of Single Amino Acid Replacements in Carmo-HrTH-II

The relative importance of each amino acid of Carmo-HrTH-II was investigated via a series of analogs in which one amino acid was substituted with an alanine residue (Table 3). In this way we can make inferences about the relative importance of the side chains of the original residues in activating the stick insect AKH receptor. The substitution of an aromatic amino acid residue (i.e., Phe^4^ or Trp^8^) with Ala eliminated AKH activity (Table 3). An Ala replacement of Thr in position 3 and 5 also gave no hypertrehalosemic effect, while a very small increase in carbohydrates was observed after injection of an Ala^7^ analog (thus, replacing Asn^7^). Ala^6^ and Ala^9^ replacements of Pro and Gly, respectively (Table 3) resulted in a marked reduction of biological activity (around 30%), whereas Ala^10^-Carmo-HrTH-II (in place of Thr^10^) resulted in about half of the maximal possible hypertrehalosemic effect. In contrast, Ala^2^-Carmo-HrTH-II increased the hemolymph carbohydrates to the same extent as did Carmo-HrTH-II (Table 3).

### Effect of Endogenous Neuropeptides and Synthetic Analogs of Carmo-HrTH-II on the Heart Rate of *C. morosus*

Adult Indian stick insects without a neck ligature are unable to respond with an increased heart beat rate upon the application of various doses of Carmo-HrTH-II (see Marco et al., 2018). In neck-ligated stick insects, however, the application of 20 pmol of the endogenous AKH peptides (Carmo-HrTH-I and -II) on the heart preparation increases the heart rate significantly from 41 beats min^–1^ to 53 beats min^1^ (*p* < 0.05; Table 4). The modified Carmo-HrTH-II with the acetylated Ala residue at the N-terminus did not significantly alter the rate of heart beat, whereas the heart rate increased (58% of the maximal response possible) after the application of the free acid-Carmo-HrTH-II and the Leu^1^ nonapeptide (Table 4). The *post hoc* test revealed that there was no significant difference between the potencies of Carmo-HrTH-I and -II (*p* > 0.05) but there was a significant difference between the result of Carmo-HrTH-II and those of the terminal modified analogs (Table 4).

**TABLE 4:** The biological effect (myotropic) of the termini of Carmo-HrTH-II in ligated 6th instar Indian stick insects (*Carausius morosus*).

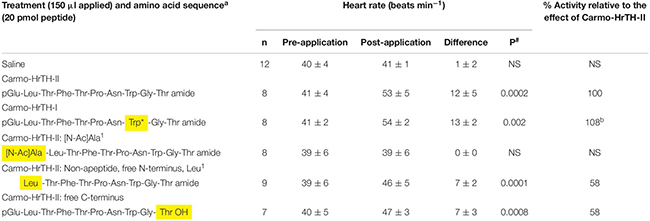

^*a*^Amino acid substitutions in Carmo-HrTH-II analogs are highlighted. *Tryptophan residue is mannosylated. ^*b*^Not significantly different from the effect of Carmo-HrTH-II (which was set as 100%), as determined by ANOVA and post hoc Scheffe’s test. The data are presented as Mean ± S.D. ^#^Paired t-test was used to calculate the significance between pre-and post-injection values. NS, not significant.

Of the bioanalogs tested, the increase in heart rate caused by the decapeptides Rommi-CC and Phyle-CC was highly significant and did not differ significantly from that caused by Carmo-HrTH-II (*p* > 0.05).

None of the systematically altered Ala analogs of Carmo-HrTH-II and none of the octapeptidic bioanalogs tested (Tables 5, 6) increased the heart rate significantly, except seemingly Ala^3^-Carmo-HrTH-II (Table 6). However, one-way ANOVA followed by the *post hoc* Scheffe’s test revealed that the response to Ala^3^-Carmo-HrTH-II is not significantly different from those of the rest of the Ala-analogs nor does it differ from the saline effect (*p* > 0.05). The data from all the systematically altered analogs and saline differed significantly from that of Carmo-HrTH-II (*p* < 0.0005).

**TABLE 5:** The biological effect (myotropic) of selected AKH bioanalogs in ligated 6th instar Indian stick insects (*Carausius morosus*).

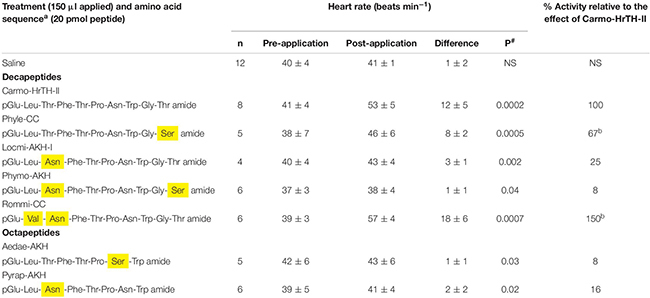

*^*a*^Amino acid substitutions in Carmo-HrTH-II analogs are highlighted. ^*b*^Not significantly different from the effect of Carmo-HrTH-II (which was set as 100%), as determined by ANOVA and post hoc Scheffe’s test. The data are presented as Mean ± S.D. ^#^Paired t-test was used to calculate the significance between pre-and post-injection values. NS, not significant.*

**TABLE 6:** The biological effect (myotropic) of single amino acid substitutions in Carmo-HrTH-II in ligated 6th instar Indian stick insects (*Carausius morosus*).

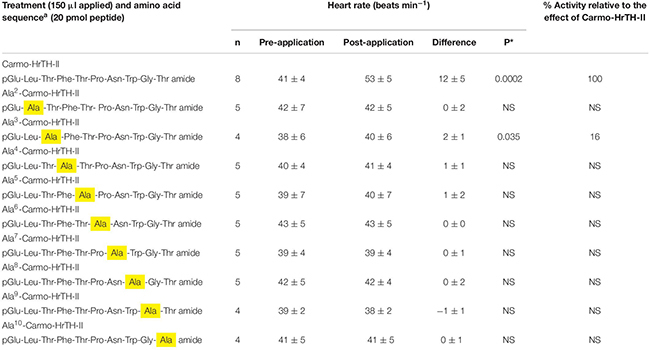

^*a*^Amino acid substitutions in Carmo-HrTH-II analogs are highlighted. Data presented as Mean ± S.D. *Paired t-test was used to calculate the significance between pre-and post-injection values. NS, not significant.

## Discussion

The peptides of the AKH/RPCH family are mainly known for their involvement in the regulation of energy metabolism, specifically the mobilization of stored fuel metabolites (lipid, carbohydrates or proline), although the AKHs are pleiotropic (Gäde, 1997; Marco and Gäde, 2020). In the stick insect, *Carausius morosus*, the endogenous members of the AKH/RPCH family act as hypertrehalosemic hormones (HrTHs) and have a stimulatory effect on heart contraction under certain conditions. The present study, thus, aimed to investigate the importance of structural features of the native HrTHs necessary to interact with the receptor on the fat body and on dorsal vessel cells of *C. morosus* to trigger a response that culminate in the release of carbohydrates into the hemolymph, and an increase in heart rate. Additionally, we were interested in whether the receptor features of *C. morosus* would more or less mirror those of the cockroach *B. discoidalis* – the only insect with a sole decapeptide AKH that had been investigated via SAR studies to date.

### Hypertrehalosemic Response in *C. morosus*

Pioneering metabolic studies with *C. morosus* (Gäde and Lohr, 1982) had revealed that the concentration of total hemolymph carbohydrates and the amount of fat body glycogen at the end of the 6th instar stage were usually higher than in adults, and that ligated stick insects show a clear and consistent hypertrehalosemic response when about to molt into adults (i.e., late-6th instar stage). The early study also demonstrated unequivocally that the ligature itself had no impact on the concentration of carbohydrates in the body over the measured period (Gäde and Lohr, 1982). The present study, thus, used carefully staged 6th instar *C. morosus* specimens to confirm that, indeed, a maximal hypertrehalosemic response was attained upon injection of a crude extract of conspecific corpora cardiaca, or the endogenous AKH members, Carmo-HrTH-I and -II. Once this was ascertained, we performed structure-activity response (SAR) studies to investigate how specific changes to the primary structure of Carmo-HrTH-II affected biological activity, and by inference, learn more about the ligand-receptor interactions in the stick insect using biological assays. The average carbohydrate concentration in the hemolymph of ligated *C. morosus* 6th instars before injection is calculated at 9.35 ± 0.96 mg/ml (*n* = 363) in the present study, which is in the same range as measured in Gäde and Lohr (1982).

Since *C. morosus* produces two functioning AKH peptides in its CC, and these decapeptides are identical in the primary amino acid sequence, we predict that the AKH receptor in this stick insect species may show a low tolerance for accommodating differences in peptide chain length (e.g., octapeptides and nona-peptides), as well as for particular amino acid substitutions in the peptide chain. The opposite was shown to be the case in the moth species, *Hippotion eson* that produces and reacts positively to five endogenous AKHs of varying chain length and sequence (Marco and Gäde, 2015, 2019).

One of the characteristic features of AKH peptides are their blocked termini: a pyroglutamic acid (pGlu) in position 1, and an amidated C-terminus, which renders protection to the ligand from exopeptidases in the hemolymph of the insect, and therefore results in a longer half-life of the peptide to achieve its hormonal effect. The current study confirms the importance of the N-terminal pGlu and C-terminal amide of Carmo-HrTH-II, the lead peptide: the terminally modified analogs showed only a slight hypertrehalosemic activity and none at all, when the N-terminal pGlu residue was eliminated and, hence, an unprotected nonapeptide was tested. Previous *in vivo* assays had also reported reduced or no activity when one of the blocking termini residues was removed or the pGlu was substituted by other blocked amino acids (Gäde, 1990; Ziegler et al., 1991, 1998; Gäde and Hayes, 1995; Lee et al., 1997; Marco and Gäde, 2015). In addition, *in vitro* receptor binding assays on the flies *D. melanogaster* and *G. morsitans* reported a decline in activation of the expressed AKH receptor when N-terminal acetylated-Ala analogs of the native AKHs were tested, whereas 40–70% receptor activation was reported with non-amidated analogs of the native fly AKH peptides (Caers et al., 2012, 2016). Hence, it is most likely that the loss of biological activity in the current study is because the deaminated analog and the analog missing pGlu were not protected from amino- or carboxypeptidases in the hemolymph of *C. morosus* which may have resulted in the peptides being partially digested before reaching the receptor. The lack of relevant activity with the N-terminal acetylated-Ala analogs *in vivo* (current study) and *in vitro* (Caers et al., 2012, 2016) suggests that the conformation of the resulting peptide differs considerably from the native conformation with pGlu, preventing proper binding to the AKH receptor. The interaction of pGlu with the AKH receptor has been shown to occur in the model for AKH ligand-receptor binding in the desert locust, *Schistocerca gregaria* (Jackson et al., 2019). Moreover, the model for the RPCH receptor of the water flea, *Daphnia pulex*, also suggests that both termini of this octapeptide are involved in binding, i.e., to the extracellular part (Jackson et al., 2018).

In addition to the termini, the chain length of the ligand appears to be critical for the *C. morosus* AKH receptor: Peram-CAH-II, one of the octapeptide bioanalogs tested in the present study, is identical to the first 8 amino acids of the lead peptide, Carmo-HrTH-II, and yet it is not able to achieve a potent hypertrehalosemic response; two other octapeptides with one amino acid substitution relative to Peram-CAH-II failed completely to achieve hypertrehalosemia (see Table 2). The response of *C. morosus* to Peram-CAH-II is in agreement with a previous study (Gäde and Lohr, 1982) that tested the CC extract of *P. americana* (thus, containing Peram-CAH-II and also the octapeptide Peram-CAH-I) in the same stick insect bioassay. The poor or no biological activity suggests that the AKH receptor of *C. morosus* has a weak affinity for octapeptides and that the two amino acids (Thr and Gly) at the C-terminal of the native Carmo-HrTH peptides are needed for receptor interaction. This is reminiscent of the situation in *B. discoidalis* where octapeptides that differ from the endogenous Bladi-HrTH only by one amino acid (besides the amino acids Gly-Thr at positions 9 and 10, of course) are more than 30-fold less active than Bladi-HrTH (Hayes and Keeley, 1990). The relevance of AKH peptide chain length does not seem to be important in an insect like *S. gregaria*, where two octapeptides and a decapeptide (Locmi-AKH-I) are endogenous AKHs. The solution structure of these three *S. gregaria* AKH peptides and models of their binding to the endogenous locust receptor were recently reported (Jackson et al., 2019), wherein it was demonstrated that all three AKHs have the same binding site on the *S. gregaria* AKH receptor, interact with similar residues of the receptor and have comparable binding constants. Now that the *C. morosus* AKH receptor is reportedly known from RNA sequencing and a *de novo* transcriptome assembly (Duan Şahbaz and Birgül Iyison, 2019), it opens the way for future *in vitro* receptor assays and molecular modeling of ligand-receptor binding to understand why (if, indeed, at all) decapeptides seem to be favored by the *C. morosus* AKH receptor. As for the decapeptide AKH bioanalogs tested in the current study, Phyle-CC with Ser^10^ instead of Thr^10^ showed the same potency as Carmo-HrTH-II (Table 2) suggesting that Thr and Ser are interchangeable because both are polar amino acids with hydroxylated side chains. The other decapeptide bioanalog with a single amino acid replacement that was tested in the current study is Locmi-AKH-I with Asn^3^ instead of Thr^3^; unlike Phyle-CC, Locmi-AKH-I could only slightly activate the *C. morosus* AKH receptor. Taking this result together with the fact that Phymo-AKH, with the double amino acid substitution of Asn^3^ and Ser^10^ instead of the endogenous Thr^3^ and Thr^10^ residues, had no significant hypertrehalosemic effect (Table 2), it is concluded that the Asn residue in position 3 is detrimental for ligand-receptor interaction. Even though both amino acids are hydrophilic, the difference is the hydroxylated side chain (Thr), whereas the second carboxy group of Asn is “neutralized” by an amide formation, resulting in a carboxyamide side chain (see Marco and Gäde, 2015). The results imply that even the removal of a simple side chain, such as a hydroxyl group, can be quite crucial for ligand-receptor interaction. However, when Asn^3^ appeared in combination with Val^2^ as in Rommi-CC (instead of the endogenous Thr^3^ and Leu^2^), hypertrehalosemic action was restored to nearly 50% of maximal activity in the current study. In Rommi-CC, the hydrophilic-hydrophobic alternating pattern is maintained by this double substitution, and having established already that the single replacement of Thr^3^ with Asn^3^ results in little receptor activation (Locmi-AKH-I in the present study), these results could mean that the long side chain of Val^2^ (as compared to Leu^2^) may be the reason for restored activity. Gäde (1992) reported that Leu and Val at position 2 of Peram-CAH-II can be interchanged without affecting the affinity of the peptide for the AKH receptor in *P. americana*. This seems to be the case with the *C. morosus* AKH receptor too, although we have not tested an AKH analog with a single replacement of Val^2^ for Leu^2^ in the current study. The lack of biological response with Locmi-AKH-I in the current study is in agreement with those of Gäde and Lohr (1982), and in another case study with the stick insect *B. extradentatum* (that also has Carmo-HrTH-II as an endogenous peptide), where Locmi-AKH-I too effected a much reduced hypertrehalosemic response in comparison to Carmo-HrTH-II (Malik et al., 2012). The replacement of Thr^3^ with Asn^3^ in a peptide tested on the moth, *H. eson*, similarly resulted in no biological activity – it should be added that Thr^3^ is conserved in all five endogenous AKH peptides of this moth (Marco and Gäde, 2015). Thus, clearly the AKH receptors of different species behave differently and it appears that co-evolution between endogenous peptide(s) and receptor has occurred for the “best fit.”

It is clear from the bioassay results with various AKH bioanalogs that side chains and charge of amino acid residues are important for an effective ligand and a biological response. Hence, in order to gain greater clarity, we designed an Ala-replacement series of analogs in the current study starting with Ala^2^ all the way to Ala^10^ to see how important side chain and charge is for the AKH activity of Carmo-HrTH-II in *C. morosus*. Ala was selected as substitution because this is a non-polar amino acid and it lacks a hydroxylated side chain. Given that Thr has a hydroxylated side chain, it is perhaps not a surprise that in this study, the single replacement of Thr at position 3, 5, or 10 of Carmo-HrTH-II, resulted in a complete loss of or a decline in biological activity (Table 3). A contributing factor for the loss of activity is that the substitution of Thr with Ala at position 3 and 5 disrupts the alternating hydrophilic-hydrophobic amino acid pattern of Carmo-HrTH-II (Thr^3^, Thr^5^, Asn^7^), which in turn might interrupt peptide conformation and thus affect the binding efficacy of the peptide. Previous studies that used *in vitro* receptor binding assays with expressed AKH receptors of dipteran insects and Ala-replacement analogs of conspecific AKHs that are similar to Carmo-HrTH-II at position 1–4 (Drome-AKH and Glomo-AKH), also showed a lack of receptor activation by analogs where Thr is substituted by an Ala residue (Caers et al., 2012, 2016). Carmo-HrTH-II has two hydrophobic amino acid residues at position 8 and 9 (Trp^8^-Gly^9^) followed by a hydrophilic Thr in position 10; thus, replacing Thr^10^ with Ala^10^ in the current study increases the C-terminal hydrophobicity of Carmo-HrTH-II, but apparently this is not so crucial as breaking up the alternating hydrophilic-hydrophobic amino acid pattern preceding this hydrophobic tail, for about 50% activity was recorded with the Ala^10^ analog of Carmo-HrTH-II (Table 3). This might also mean that the hydroxyl group present on the terminal end (Thr^10^) is less important for peptide-receptor interaction in *C. morosus* compared with that present on non-terminal residues (Thr^3^, Thr^5^). In a study with modifications of the Thr residues in Locmi-AKH-I, Poulos et al. (1994) demonstrated that in *L. migratoria*, the hydroxyl group of the Thr^5^ of Locmi-AKH-I is important for biological activity, while that of Thr^10^ is not. Similarly, in *B. discoidalis* a single amino acid change at position 10 was well tolerated without much loss in bioactivity (Ford et al., 1988).

The current study revealed that the substitution of Leu^2^ in Carmo-HrTH-II with Ala has no impact on the AKH receptor on the fat body of *C. morosus*. Both Leu and Ala residues are non-polar. The only difference between these residues is that Leu has a bulkier alkyl side chain than Ala [-CH_2_CH(CH_3_)_2_ vs. -CH_3_]. This might mean that only one methyl group of Leu is involved in the receptor interaction or the whole side chain may, indeed, not be so important. Similarly, in *B. discoidalis* Ala in position 2, instead of Val in the endogenous Bladi-HrTH, can also be tolerated well (Ford et al., 1988). Finally, two aromatic amino acids are conserved in AKHs and form part of the hallmark features of the AKH peptide family; these are Phe^4^ and Trp^8^. Not surprisingly, therefore, replacement of the aromatic side chains with Ala in the present study resulted in the complete loss of metabolic activity in *C. morosus*, as has been shown for other insect species too in *in vivo* and *in vitro* receptor assays (Ford et al., 1988; Gäde and Hayes, 1995; Ziegler et al., 1998; Caers et al., 2012, 2016; Marco and Gäde, 2015), thus signifying that these structural features are crucial for receptor-binding in insects. When these aromatic amino acids were swapped to construct a Locmi-AKH-I with Trp^4^ and Phe^8^, it was not very active in *L. migratoria* (Velentza et al., 2000). Moreover, single substitutions of Phe^4^ with Trp of Locmi-AKH-I is tolerated (a 10-fold loss of potency), while the replacement of Trp^8^ with Phe is not (>300 times decrease in potency), leading to the conclusion that position 4 requires a phenyl ring in the side chain, and position 8 an indole ring (Velentza et al., 2000). Ligand interaction diagrams for the two AKH receptor models (*S. gregaria*; *D. pulex*) show also clearly that the two aromatic residues in the molecule are essential for binding (Jackson et al., 2018, 2019).

AKHs are predicted to have a β-turn at position 5–8 (Hayes and Keeley, 1990; Zubrzycki and Gäde, 1994; Cusinato et al., 1998) and, although amino acid sequences and chain length of AKHs can vary, nuclear magnetic resonance experiments assigned turns for each of the examined AKHs (see, for example, Jackson et al., 2019). If this is true for Carmo-HrTH-II as well, then the single replacement of these amino acids with Ala, which results in the removal of the side chain and/or the interruption of alternating hydrophilic pattern, might disrupt the peptide β-conformation. Interrupting the stability of the conformation may hinder the interaction of the peptide with its receptor through the backbone hydrogen bonding (Gäde and Hayes, 1995). In the current study, single replacements of amino acids at position 5–9 of Carmo-HrTH-II with Ala resulted in complete loss of potency. This is quite unique. In most studies position 7 could be replaced without major loss of activity (see, for example, Ford et al., 1988; Gäde and Hayes, 1995). In *B. discoidalis* positions 2, 7, and 10 were the ones least affected by single substitution. Unexpectedly the *C. morosus* AKH receptor did accept the change at position 2 very well (Table 3).

### Cardio-Stimulatory Activities in *C. morosus*

The effect of neuropeptides on the contraction of the dorsal heart was studied before in stick insects, including *C. morosus* (Ejaz and Lange, 2008; da Silva et al., 2011; Malik et al., 2012; Marco et al., 2018). Although members of the AKH/RPCH family are reported to stimulate the heart beat rate in insects, including stick insects that were either decapitated or ligated behind the head (see Chowański et al., 2016; Marco et al., 2018), it is not known whether these peptides act directly or indirectly on the heart. It is, nevertheless, thought that the stimulation of the heart by the AKH peptides is a mechanism for assisting the faster distribution of fuel metabolites (Gäde and Marco, 2013). In the current study we applied the same bioanalogs and analogs of Carmo-HrTH-II that were tested in *in vivo* metabolic assays also in a semi-isolated heart assay with *C. morosus* to see whether the respective biological output could lead to a conclusion about the receptor identity or receptor needs in the two physiological systems.

The application of the N- or C- terminal modified analogs on the *C. morosus* heart preparations resulted in 58% increase in the frequency of heart contractions compared to that of Carmo-HrTH-II, while the analog that had a pGlu replaced with N-acetyl-Ala did not increase the heart rate. These results indicate that the native peptide may lose some of its binding affinity when the pGlu is removed, or with a free acid at the C-terminus; presumably, the breakdown of the peptides are not as rapid in this assay where the peptides are directly applied to the heart, as opposed to the case of the metabolic assay where the peptides are in the hemolymph and exposed to exopeptidases for 90 min. Further, these results suggest that the peptide analogs with the free termini retain a conformation that can bind to the *C. morosus* AKH receptor, whereas the peptide conformation brought about through the N-acetyl-Ala in position one is not conducive for ligand-receptor binding. The metabolic and the heart assay results in the current study largely indicate the same outcome. In *P. americana*, the affinity is completely lost, with no stimulation of the heart rate when the pGlu or amide is removed in both *in vivo* and semi-isolated heart assays (Baumann et al., 1990).

Single replacements of amino acids with Ala at all positions of Carmo-HrTH-II resulted in the peptide losing its efficacy completely. The data indicate that the side chains of all the amino acids of Carmo-HrTH-II are crucial for eliciting cardio-stimulatory action in *C. morosus*. This is mostly comparable with the data from the metabolic assays *in vivo* where small metabolic changes are measured, while the heart beat is totally unresponsive to the analogs. There is, however, one anomaly that is not easily explained: the Ala^2^ analog was as active biologically as the lead peptide Carmo-HrTH-II in raising the carbohydrate concentration in the ligated stick insects (Table 3); this same analog did not have a significant effect on the heart contractions in the semi-exposed heart assay (Table 6). We are not able to explain this phenomenon at present.

The myotropic effect measured with the selection of bioanalogs yielded largely comparable results to those obtained in the *C. morosus* metabolic assay, with Rommi-CC and Phyle-CC standing out as active peptides, and only a very small effect measured with the other decapeptide analogs and the octapeptides.

Since the data trends between the two assays and the peptide analogs tested are so comparable, we conclude that the same receptor is at play in both physiological systems: the rate of heart beat and the mobilization of carbohydrates from the fat body stores in ligated stick insects.

Further studies could be carried out to investigate the mode of action of AKH/RPCH peptides in non-ligated *C. morosus* and other stick insect species since they respond with a biological effect only when the circulation is disrupted between the head and the rest of the body. Wicher et al. (2006) demonstrated that the AKHs of the cockroach *P. americana*, in addition to the metabolic function, act directly on the central nervous system through the release of octopamine from the thoracic dorsal unpaired median (DUM) neurons, and this release of octopamine stimulates locomotor activity. Octopamine is a well-known “fight-or-flight” stress hormone in insects (Verlinden et al., 2010), and its action as heart stimulant has been studied in many insect species (see Chowański et al., 2016). Indeed, direct octopaminergic innervation of the insect heart is observed in several insect species, arising from the DUM neurons, and is responsible for cardioacceleratory responses in *D. melanogaster*, *P. americana*, and *M. sexta* (see Johnson et al., 1997; Zornik et al., 1999; Papaefthimiou and Theophilidis, 2011). In *C. morosus*, however, octopamine unequivocally inhibits the contraction of the heart of *C. morosus* in a dose-dependent manner, and this is interpreted as an appropriate response in an insect species that relies on cryptic biology to escape predators (Marco et al., 2018). *C. morosus* masquerades as a stick or twig in its habitat, engages in slow movements to keep up the pretense of being part of the food plant, and escapes the interest of predators through thanatosis (i.e., playing dead when detected; Gullan and Cranston, 2014). The Indian stick insect, thus, relies on a strategy of concealment (low energetic costs) in which fight, flight and energy mobilization play no role. By extension, it is expected that the role of stress hormones, such as octopamine and AKHs, would have an opposite physiological effect in such a species.

### Lessons for Green Insecticide Design

Phasmids are generally not regarded as serious pest insects since they do not pose a direct threat to food security, nor are they known to be vectors of diseases, nevertheless quite damaging outbreaks of stick insect population numbers have been recorded over the years in Australia, North America, China and other geographical areas, where they defoliate economically important timber crops (Baker, 2015). Such defoliated timber trees respond in subsequent years with a smaller stem diameter which is deleterious to the pulp industry. Although many stick insect species are apterous and can therefore not spread as rapidly and widely as winged insects, they can have a serious local impact in the event of an outbreak (Baker, 2015). Pest status notwithstanding, are there any lessons to learn from our work here on the physiological action of AKH ligands in the Indian stick insect *C. morosus* that may be useful for the design of a peptide mimetic that could act specifically to target known pest insects without interfering with other insects?

To date, only a small number of stick insect species have been studied with respect to their AKH neuropeptides. Besides *C. morosus*, primary structures are known from *Sipyloidea sipylus*, *Extatosoma tiaratum*, and *Baculum extradentatus* which all synthesize the decapeptide Carmo-HrTH-II (pELTFTPNWGTa) in their CC as the major AKH family neuropeptide, as well as a less abundant neuropeptide that is also biologically active (Gäde, 1989; Gäde and Rinehart, 1990; Malik et al., 2012). In the case of *C. morosus* and *B. extradentatus*, the less abundant peptide was characterized as a post-translational variant of Carmo-HrTH-II, viz. at position 8 the Trp is C-mannosylated (Carmo-HrTH-I, Munte et al., 2008) or modified to kynurenine (Malik et al., 2012). While the structural identity of the additional neuropeptide in *S. sipylus* and *E. tiaratum* was not pursued (due to a lack of sufficient material at the time), we speculate that it may also be Carmo-HrTH-II with a post-translationally modified Trp, and this may be a trait in other stick insects with Carmo-HrTH-II. Recent genomic data sets (Veenstra, 2019) revealed that Carmo-HrTH-II is encoded in the New Zealand stick insect *Clitarchus hookeri* whereas in the evolutionary basal stick insect *Timema christinae* an octapeptide (pEVNFSPSWa) is encoded; this octapeptide is well-known as Anaim-AKH and found in certain dragonflies (Gäde and Marco, 2005) and other basal orders of insects such as Archaeognatha (Marco et al., 2014) and Ephemeroptera (Gäde and Marco, 2012). Carmo-HrTH-II and Anaim-AKH are structurally vastly different peptides – not only in sequence length but also in the actual amino acid sequence. To base a putative lead peptide for AKH insecticide use on the octapeptide would certainly also affect dragonflies which are mostly endangered species, whereas it is envisaged that *C. morosus* will not be targeted by most AKH mimetics as pesticides because of the very specific needs required for ligand-receptor binding in this species, as deduced here from biological assays: the AKH receptor of *C. morosus* accepts decapeptides and only the amino acids in positions 2 and 10 may deviate from the Carmo-HrTH-II primary sequence.

This seem to be good criteria for specificity but what do we know about the effect of Carmo-HrTH-II on other insects? SAR data only exist from *in vivo* assays and they revealed the following:

(1) Carmo-HrTH-II was more than 300-fold less active in the cockroach *B. discoidalis* than the endogenous Bladi-HrTH (Hayes and Keeley, 1990).
(2) Carmo-HrTH-II was as, or slightly more active than the endogenous nonapeptide Manse-AKH in the lepidopteran *Manduca sexta* (Fox and Reynolds, 1991; Ziegler et al., 1991).
(3) Carmo-HrTH-II was more or less as active in *P. americana* as the endogenous octapeptides Peram-CAH-I and -II (Gäde, 1990).
(4) Carmo-HrTH-II was only slightly less active in *L. migratoria* than the endogenous decapeptide Locmi-AKH-I (Gäde, 1990).

Hence, the effect of an insecticidal peptide based on Carmo-HrTH-II would very likely also be effective against some serious pest insects such as a number of lepidopteran larvae, migratory locusts and blattid cockroaches which is not undesirable.

## Data Availability Statement

All datasets generated for this study are included in the article/supplementary material.

## Author Contributions

HM and GG conceptualized the research project, financed the work, and supervised OK. OK and HM performed experiments, reared the animal cultures, and analyzed the data. GG contributed chemicals and peptides, helped with interpretation of the data, and writing the draft manuscript. OK performed most biological assays, was involved in data interpretation, and drafting of the manuscript. HM helped with interpretation and analyses of data, writing, and refining the draft manuscript. All the authors agreed to be accountable for the content of the work presented here.

## Conflict of Interest

The authors declare that the research was conducted in the absence of any commercial or financial relationships that could be construed as a potential conflict of interest.
